# Surface Layer Protein A Variant of *Clostridium difficile* PCR-Ribotype 027

**DOI:** 10.3201/eid1702.100355

**Published:** 2011-02

**Authors:** Patrizia Spigaglia, Fabrizio Barbanti, Paola Mastrantonio

**Affiliations:** Author affiliations: Istituto Superiore di Sanità, Rome, Italy

**Keywords:** Clostridium difficile, S-layer, surface layer protein A, slpA, virulence, enteric infections, antimicrobial resistance, bacteria, letter

To the Editor: Rates and severity of *Clostridium difficile* infection (CDI) have recently increased worldwide and correlate with dissemination of hypervirulent epidemic strains designated PCR-ribotype 027. CDI caused by this PCR-ribotype is characterized by strong toxin A and B production, presence of binary toxin genes, and, usually, a high level of resistance to fluoroquinolones (*1*).

The mechanisms by which *C. difficile* colonizes the gut during infection are poorly understood. In addition to the toxins, surface protein components are undoubtedly involved. In particular, the surface layer (S-layer) mediates adhesion to enteric cells (*2*), but other functions have been proposed for this S-layer structure: it may act as a molecular sieve, protect against parasitic attack, or be a mechanism to evade the host immune system (*3*). Furthermore, the *C. difficile* S-layer is the predominant surface antigen and is among the main potential candidates for multicomponent vaccines against CDI (*4**,**5*). Composed of 2 major components, the *C. difficile* S-layer has high and low molecular weight proteins (HMW and LMW, respectively), which are formed from the posttranslational cleavage of a single precursor, surface layer protein A (slpA) (*6*). Different variants of the *slp*A gene have been identified in *C. difficile* (*7*).

The complete genome sequences of 2 *C. difficile* PCR-ribotype 027 strains (CD196, a nonepidemic strain isolated in France in 1985, and R20291, isolated from an outbreak in Stoke Mandeville, UK, in 2006) have been recently deposited in GenBank (accession nos. FN538970 and FN545816, respectively) (*8*). We analyzed the *slp*A gene of these strains by using the National Center for Biotechnology Information BLAST server (www.ncbi.nlm.nih.gov/blast)and the European Bioinformatics Institute ClustalW server (www.ebi.ac.uk/clustalw). Both strains showed a new and identical *slpA* nucleotide sequence. To determine if the new variant was conserved among PCR-ribotype 027 strains, we characterized 8 additional epidemic strains belonging to this PCR-ribotype that were isolated in different geographic regions and years and showed different patterns of resistance to erythromycin and moxifloxacin. Three strains, AI13, AII6, and AIII8, were isolated in 3 hospitals in Belgium during a European prospective study conducted in 2005 (*9*). *C. difficile* DI12 was isolated in Ireland during the same study. *C. difficile* GII7 and LUMC46 were isolated in the Netherlands in 2005 and 2008, respectively. *C. difficile* M43 and A422 were isolated in Calgary (Canada) in 2001 from 2 outbreaks.

Six strains were resistant to erythromycin (MICs >256 mg/L) and moxifloxacin (MICs 12–256 mg/L). AIII8 was resistant to erythromycin (MIC >256 mg/L) and intermediately resistant moxifloxacin (MIC = 6 mg/L), whereas CD196, LUMC46, and A422 were susceptible to both drugs.

The *slp*A genes of all strains were amplified by PCR mapping. Nine primers were designed on the *slpA* region to obtain 10 overlapping PCR products. The positions of the primers on the reference sequence FN545816 were 3161991–31612012, 3162346–3162365, 3162728–3162746, 3162746–3162728, 3163514–3163495, 3164222–3164205, 3163264–3163284, 3163284–3163264, and 3164518–3164499. Target amplification was performed by an initial denaturation at 94°C for 5 min, then 30 cycles of 94°C for 1 min, 50°C for 1 min, and 72°C for 1 min. Sequence assembly was performed by using DNAStar Lasergene version 8.0 software (DNAStar, Madison, WI, USA). The protein analysis was performed by using the SignalP 3.0 server (www.cbs.dtu.dk/services/SignalP/) and the ExPASy Proteomics server (www.expasy.ch/tools/pi_tool.html). Amino acid comparisons were accomplished by using ClustalW (www.ebi.ac.uk/clustalw), and the output was used for construction of the phylogenetic tree by TreeView version 1.6.6 (http://en.bio-soft.net/tree/TreeView.html). All PCR-ribotype 027 strains showed the same *slp*A gene nucleotide sequence. The slpA precursor encoded by this gene contained a signal peptide, and its cleavage site was located between aa 24 and aa 25. The cleavage of the slpA precursor into LMW and HMW proteins was predicted between aa 342 and aa 343 (N terminal to an Ala amino acid residue and C-terminal to a consensus motif Thr-Lys-Ser). The molecular masses of the LMW and HMW proteins were 33.871 kDa and 44.174 kDa, respectively. These protein sizes were confirmed by sodium dodecyl sulfate polyacrylamide gel electrophoresis, after a low pH glycine extraction (data not shown). The phylogenetic tree (Figure), obtained by comparison with the amino acid sequences of other PCR ribotypes (*6*), showed that *C. difficile* strain 027 slpA was strongly related (identity 89%) to that of strains belonging to the epidemic PCR-ribotype 001. In particular, the identity between the 2 PCR ribotypes was 100% for the HMW proteins and 77% for the LMW proteins.

**Figure:**
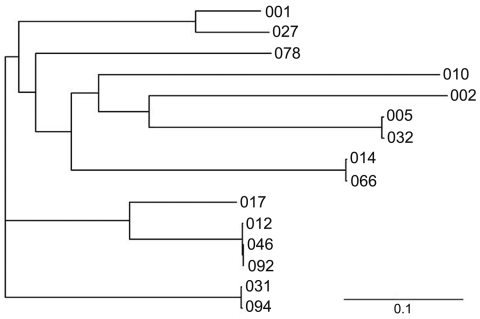
Phylogenetic tree based on the alignment of the surface layer protein A amino acid sequence of *Clostridium difficile* 027 (GenBank accession no. CBE06198) with those of PCR-ribotypes 001, 002, 005, 010, 012, 014, 017, 031, 046, 054, 066, 078, 092, and 094 (GenBank accession nos. AAZ05957, AAZ05964, AAZ05968, AAZ05974, AAZ05975, AAZ05984, AAZ05988, AAZ05989, AAZ05980, AAZ05972, AAZ05986, AAZ05994, AAZ05982, and AAZ05991, respectively). The phylogram was generated by using TreeView version 1.6.6 (http://en.bio-soft.net/tree/TreeView.html). The branch lengths are scaled in proportion to the extent of the change per position, as indicated by the scale bar.

This study provides convincing evidence that the S-layer is well conserved in *C. difficile* PCR-ribotype 027 strains and has high identity with the slpA of the epidemic PCR-ribotype 001. Because *C. difficile* PCR-ribotypes 027 and 001 are the most frequently isolated strains from severe CDIs across both North America and Europe (*9**,**10*), the result obtained suggests that the S-layer of these virulent strains presents peculiar and common characteristics that could be an advantage for these bacteria during the infection process.
